# Conversion of Pectin-Containing By-Products to Pectinases by *Bacillus amyloliquefaciens* and Its Applications on Hydrolyzing Banana Peels for Prebiotics Production

**DOI:** 10.3390/polym13091483

**Published:** 2021-05-04

**Authors:** Chien Thang Doan, Chien-Lin Chen, Van Bon Nguyen, Thi Ngoc Tran, Anh Dzung Nguyen, San-Lang Wang

**Affiliations:** 1 Department of Chemistry, Tamkang University, New Taipei City 25137, Taiwan; dcthang@ttn.edu.vn (C.T.D.); vic900065@gmail.com (C.-L.C.); ttngoc@ttn.edu.vn (T.N.T.); 2 Faculty of Natural Sciences and Technology, Tay Nguyen University, Buon Ma Thuot 630000, Vietnam; 3 Institute of Biotechnology and Environment, Tay Nguyen University, Buon Ma Thuot 630000, Vietnam; nvbon@ttn.edu.vn (V.B.N.); nadzung@ttn.edu.vn (A.D.N.); 4 Life Science Development Center, Tamkang University, New Taipei City 25137, Taiwan

**Keywords:** pectin, pectinase, wheat bran, banana peel, *Bacillus amyloliquefaciens*, prebiotics

## Abstract

The utilization of pectin-containing by-products may be useful in a variety of fields. This study aims to establish the processing of pectin-containing by-products to produce pectinases using *Bacillus amyloliquefaciens* TKU050 strain. In this study, several kinds of agricultural pectin-containing by-products from banana (banana peel), rice (rice bran), orange (orange peel), coffee (spent coffee grounds), and wheat (wheat bran) were utilized to provide carbon sources for the production of a pectinase by *B. amyloliquefaciens* TKU050. *B. amyloliquefaciens* TKU050 expressed the highest pectinase productivity (0.76 U/mL) on 0.5% wheat bran-containing medium at 37°C for four days. A 58 kDa pectinase was purified from the four-day cultured medium fermented under optimized culture conditions with 7.24% of a recovery ratio and 0.51 U/mg of specific activity, respectively. The optimum temperature, optimum pH, thermal stability, and pH stability of the TKU050 pectinase were 50 °C, pH 6, <50 °C, and pH 6–9, respectively. The TKU050 pectinase was inhibited by sodium dodecyl sulfate and Cu^2+^. The reducing sugar obtained by hydrolyzing banana peel with TKU050 pectinase showed the growth-enhancing effect on the growth of four tested lactic acid bacteria.

## 1. Introduction

Pectins are a wide category of polysaccharides that consist mainly of galacturonic acid as well as some L-arabinose, D-galactose, and L-rhamnose units that can be split into three major groups, including homogalacturonan, rhamnogalacturonan-I, and rhamnogalacturonan-II [1]. In nature, pectins are found abundantly in higher plants’ primary cell walls and middle lamella [2,3]. The main enzymes responsible for pectin degradation are pectinases [4]. There are three categories of pectinases based on their action mode and enzymatic substrate, including polygalacturonases, lyases, and pectin methylesterases [2].

According to studies, microbial pectinase accounts for 25% of global food and industrial enzyme revenues, and the demand is constantly expanding [5]. *Penicillium* spp., *Aspergillus* spp., *Erwinia* spp., and *Bacillus* spp. are among the microbial strains that have been commonly used in the commercial development of pectinases. [2]. However, many of those researches used commercial pectin as the carbon source for pectinase production [2,6]. Consequently, the high cost of pectin could raise the price of enzyme production, thereby limiting pectinase’s applications. This problem can be partially solved by using agro-waste sources, such as citrus peel [7], wheat bran [8], apple pomace [9], grape pomace [10], papaya peel [9], or banana peel [11], etc., as alternative substrates for the microbial fermentation process. Additionally, bacterial pectinase is favored over fungal pectinase due to its ease of fermentation and modern techniques for increasing production yield [12].

Pectinases are essential in a variety of industries. In the food and beverage industries, they are a useful tool for making jams and jellies [2], clarifying wine [13], extracting juice [14] and vegetable oil [15], removing mucilage from coffee beans [16], concentrating and fermenting tea, cocoa, and coffee, and pickling [17]. In the paper and textile industries, pectinases are used for bleaching pulp, bio-scouring cotton, retting, and degumming plant fibers [2]. Pectinases are also useful in the processing of bioenergy, poultry feed, wastewater treatment, and protoplast fusion technology [2,17].

The search for novel enzymes with a low-cost production process and appropriate properties is considered critical research. Thus, this study aims to produce pectinases by *B. licheniformis* TKU050 using pectin-containing by-products as the carbon source. This can simultaneously achieve two objectives: lowers the cost of enzyme production and reducing the amount of waste generated in the environment. Accordingly, various kinds of pectin-containing byproducts, such as wheat bran, rice bran, banana peel, pomelo peel, spent coffee grounds, and orange peel, have been used as carbon sources for *B. amyloliquefaciens* TKU050 to produce pectinase. The properties of pectinase, as well as its purification, have been studied. Furthermore, among several kinds of pectin-containing by-products, banana peel was observed as the most suitable substrate for *B. amyloliquefaciens* TKU050 pectinase. Banana peel is a well-known agricultural waste that contains a range of nutrients and bioactivity, including antioxidants [18,19], antimicrobial [20], anti-cancer [21], and enhance α-glucosidase inhibitory in yogurt [22]. Banana peel makes up about 40% of the fruit weight, and the recovery of banana peel for its bioactivity compounds is an essential step toward agricultural sustainability. However, there is no relevant report on its prebiotic effects after being hydrolyzed by pectinases. Therefore, the growth-enhancing effects of the reducing sugar obtained by hydrolyzing with TKU050 pectinase on lactic acid bacteria were also investigated.

## 2. Materials and Methods

### 2.1. Materials

*Bacillus amyloliquefaciens* TKU050 was isolated from the soil using pectin as the sole carbon source. Pectin was bought from Acros Organics (Fisher Scientific, Göteborg, Sweden). *Lactococcus lactis* subsp. *lactis* BCRC10791, *Lactobacillus rhamnosus* BCRC104940, *Lactobacillus paracasei* subsp. *paracasei* BCRC14023, and *Lactobacillus rhamnosus* BCRC16000 were obtained from the Bioresource Collection and Research Center (Hsinchu, Taiwan). The other chemicals used were of the highest possible quality.

### 2.2. Screening, Selection, and Identification of the Pectinase-Producing Bacterium

A pectin-containing medium (1% (*w*/*v*) pectin, 0.2% K_2_HPO_4_, 0.2% KH_2_PO_4_, 0.2% KNO_3_, and 0.3% yeast extract) was used to isolate the pectinase-producing bacteria. Bacterial strains collected from Tamkang University (New Taipei, Taiwan) soil were incubated in a pectin-containing agar medium for three days at 37 °C and a 150-rpm shaking speed, after which the supernatant was withdrawn to examine its pectinase activity. Later on, the selected strain was characterized, and its scientific name was identified by morphological, API 50 CHB/E kit, and 16S rDNA sequences analysis.

### 2.3. Pectinase Assay

The dinitrosalicylic acid (DNS) method [23] was used to determine the amount of reducing sugars produced from the pectin hydrolysis reaction catalyzed by pectinase. A pectin hydrolysis reaction was carried out with an equivalent volume of substrate (0.2 mL of 1% *w*/*v* pectin) and diluted sample (0.2 mL) with 30 min of reaction time and an incubation temperature of 37 °C. Next, 1.5 mL of DNS reagent was introduced to the mixture, which was then heated for 10 min at 100 °C. After centrifugation (4000× *g*, 10 min), the color of the reaction was measure by an ELISA plate reader to calculate the pectinase of the sample. A pectinase unit is the volume of enzyme needed to produce 1 µmol of D-galacturonic acid in one minute.

### 2.4. By-Products of Agriculture Processing as the Sole C/N Source for Pectinase Production

To prepare a medium containing agriculture by-products (ABP), 1% of each ABP (wheat bran, rice bran, banana peel powder, orange peel powder, coffee grounds, and pectin) was added into 100 mL of a basal medium containing 0.2% K_2_HPO_4_, 0.2% KH_2_PO_4_, 0.2% KNO_3_, and 0.3% yeast extract. The fermentation of *B. amyloliquefaciens* TKU050 on different ABP-containing mediums was initiated by adding 1 mL of bacterial seed solution to 100 mL of each medium and then incubating the solution at 37 °C and a 150-rpm shaking speed. During the cultivation period, the pectinase activity of the growth media was tested every 24 h. Some wheat bran powder (WBP) amounts (0.5, 1.0, 1.5, and 2.0 g) were added to 100 mL of basal medium to explore the optimal wheat bran concentration for *B. amyloliquefaciens* TKU050 pectinase synthesis. The fermentation conditions have already been mentioned in this study.

### 2.5. Enzyme Purification

*B. amyloliquefaciens* TKU050 was incubated as per the optimal conditions for pectinase production. The culture broth was centrifuged at 4000× *g* for 10 min to remove the residual solids and bacterial cells. The liquid supernatant was then mixed with ammonium sulfate (80% *w*/*v*) and kept at 4 °C for one night. The precipitate that appeared in the culture supernatant was conveniently obtained by centrifugation (10,000× *g*, 20 min). A small volume of 50 mM NPB at pH 7 was used to dissolve the obtained precipitate. The residual ammonium sulfate in the crude supernatant was removed by dialyzing the solution against NPB for one night using a cellulose membrane (cellusep T2, Interchim, Montluçon, France). For the enzyme purification, the crude enzyme was loaded onto a Macro Prep High Q column (Bio-rad, Hercules, California, USA) pre-equilibrated with NPB (50 mM, pH 7). The washing step took place until the A280 nm reached a stable value. After the washing step, a linear gradient of NaCl (0 M–1.0 M) was conducted to elute the target enzymes. Fractions exhibiting pectinase activity were then pooled and concentrated by the lyophilization method. Finally, an HPLC system consisting of a KW-802.5 column was used to purify the obtained enzyme (a flowrate of 0.6 mL/min, an A280 nm detector, and a column temperature of 25 °C).

### 2.6. Sodium Dodecyl Sulfate-Polyacrylamide Gel Electrophoresis (SDS-PAGE) Analysis

SDS-PAGE was conducted in a 10% resolving gel according to the method described previously [24]. The sample was prepared in a sample buffer containing 2-mercaptoethanol and SDS, and the mixture was heated at 100 °C for two min. Then, a 10 µL sample/well was loaded for electrophoresis at 25 mA, after which the gel was stained by a protein assay dye reagent (Bio-Rad, Berkeley, CA, USA) and then de-stained by methanol/acetic acid/water solution (1/1/8, *v*/*v*/*v*). The visual band of the enzyme was compared with the bands of the protein markers to determine its MW.

### 2.7. Effect of pH and Temperature

By altering the pH of the reaction solution, the optimal pH of *B. amyloliquefaciens* TKU050 pectinase was determined in the same way as the pectinase activity test described above. Sodium carbonate (pH 9–11), sodium phosphate (pH 6–8), sodium acetate (pH 5), and glycine HCl (pH 2–4) made up the pH change buffer system. The pH stability of *B. amyloliquefaciens* TKU050 pectinase was calculated by measuring its residual activity after incubating the enzyme for one hour at different pH levels ranging from 2 to 11. The reaction of the enzyme and 1% pectin solution was held at various temperatures to determine the optimal temperature of the *B. amyloliquefaciens* TKU050 pectinase. The remaining activity of *B. amyloliquefaciens* TKU050 pectinase after one hour of incubation at different temperatures (10–100 °C) was the basis for determining its thermal stability. The remaining activity of *B. amyloliquefaciens* TKU050 pectinase was tested following the procedure mentioned above.

### 2.8. Substrate Specificity

The substrate specificity of *B. amyloliquefaciens* TKU050 pectinase was tested on pectin, starch, dextran, gum arabic, carboxymethyl cellulose (CMC), banana peel powder (BPP), orange peel powder, wheat bran powder, rice bran powder (RBP), pomelo peel powder (PPP), coffee pulp powder (CPP), and spent coffee grounds (SCG) using the conditions for the pectinase activity test as described above. The pectinase activity in pectin was used as the control.

### 2.9. Effect of Metal Ions, Inhibitors, and Surfactants

The chemicals tested included metal ions (Zn^2+^, Fe^2+^, Mn^2+^, Cu^2+^, Ba^2+^, and Ca^2+^), EDTA, and surfactants (SDS, triton X-100, tween 20, and tween 40). Initially, a *B. amyloliquefaciens* TKU050 pectinase solution was added to each of those chemicals in the same proportion for 30 min, and then, the residual activity of the pectinase was determined by the methods described above. 

### 2.10. Growth Enhancing Effect of BPP Oligosaccharides on Lactic Acid Bacteria Test

*Lactobacillus paracasei* subsp. *paracasei* BCRC14023, *L. rhamnosus* BCRC16000, *L. rhamnosus* BCRC10494, and *Lactobacillus lactis* BCRC10791 were used to evaluate the growth-enhancing effect of BPP hydrolysate, pectin hydrolysate, and BPP. The bacteria were cultured in an MRS medium containing 0.1% (*w*/*v*) BPP hydrolysate, pectin hydrolysate, BPP, or glucose for 48 h at 37 °C. The 655 nm optical density of the culture supernatant was used to measure the cell growth of the bacteria.

## 3. Results and Discussion

### 3.1. Screening and Identification of Strain TKU050

Over 300 bacterial strains isolated from the soils of Tamsui (New Taipei City, Taiwan) were cultivated at 37 °C in a medium containing 1% pectin. Among them, strain TKU050 exhibited the highest pectinase activity and was selected for further investigations. To characterize strain TKU050, 16S rDNA sequencing and phylogenetic analyses were performed. Based on the evaluation of the 16S rDNA gene sequence, TKU050 was most closely aligned to *Bacillus amyloliquefaciens*, with 95% similarity. To further characterize strain TKU050, standard morphological, physiological, and biochemical plate analysis showed that strain TKU050 was a Gram-positive and endospore-forming bacillus that grows in both aerobic and anaerobic environments. According to the API 50 CHB/E identification, TKU50 was most closely related to *B. amyloliquefaciens*. The phylogenetic identification and API 50 CHB/E analysis indicated that strain TKU050 belonged to the species *B. amyloliquefaciens*.

### 3.2. Effect of the Carbon (C) Source on Pectinase Production

Six types of pectin sources, including banana peel powder (BPP), orange peel powder (OPP), spent coffee grounds (SCG), wheat bran powder (WBP), rice bran powder (RBP), and pectin powder was used as the sole C source to investigate their effect on pectinase production by *B. amyloliquefaciens* TKU050. One gram of each pectin source was added to 100 mL of a basal medium containing 0.2% K_2_HPO_4_, 0.2% KH_2_PO_4_, 0.2%, KNO_3_, and 0.3% yeast extract, which was then shaken at 150 rpm for five days.

**Figure 1:**
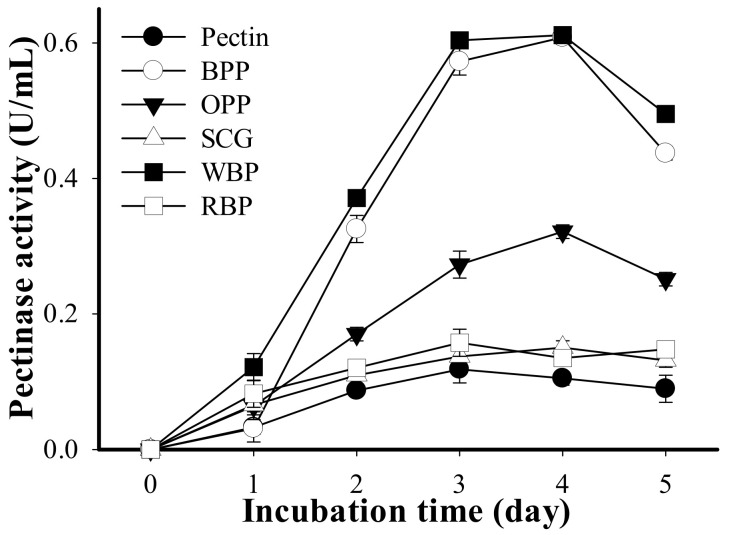
Screening of a 1% (*w*/*v*) suitable C source for pectinase production by *B. amyloliquefaciens* TKU050, including BPP (banana peel powder), OPP (orange peel powder), SCG (spent coffee grounds), WBP (wheat bran powder), and RBP (rice bran powder). All error bars are the ±SD (standard deviation) of three different experiments.

As shown in Figure 1, all five tested agricultural by-products received higher pectinase productivity than that of commercial pectin products. *B. amyloliquefaciens* TKU050 produced the highest pectinase activity in the medium containing WBP (0.612 U/mL on the fourth day of the fermentation period) and then decreased in the order of BPP (0.608 U/mL on the fourth day), OPP (0.322 U/mL on the fourth day), RBP (0.158 U/mL on the third day), SCG (0.150 U/mL on the fourth day), and pectin (0.118 U/mL on the fourth day). As such, WBP showed promise as a potential C source for pectinase production by *B. amyloliquefaciens* TKU050. Further investigation of the pectinase production of *B. amyloliquefaciens* TKU050 using the WBP-containing medium indicated that a lower concentration of WBP would be more appropriate for higher pectinase productivity. Accordingly, the highest pectinase activity was observed in the four-day medium containing 0.5% WBP (0.515 U/mL), followed by the 1% WBP (0.495 U/mL), 1.5% WBP (0.448 U/mL), and 2% WBP (0.260 U/mL). As such, 0.5% (*w*/*v*) was selected as the optimum WBP concentration for pectinase production by *B. amyloliquefaciens* TKU050.

### 3.3. Optimization of Culture Conditions for Pectinase Production

WBP (0.5%, *w*/*v*) was confirmed as a potential source of carbon and was chosen for an optimization study of a number of parameters, including cultivation pH (3–9), cultivation temperature (30, 37, 40, 45, and 50 °C), and shaking speed (50, 100, 150, and 200 rpm). The effect of cultivation pH on pectinase production was performed using a 100-rpm shaking speed, a medium/flask volume ratio of 100/250 mL (0.2% K_2_HPO_4_, 0.2% KH_2_PO_4_, 0.2% KNO_3_, and 0.3% yeast extract), and 0.5% WBP (C source). The pectinase activities produced in different pH media were all highest on the fourth day in the order of pH 6 (0.764 U/mL), pH 7 (0.722 U/mL), pH 5 (0.714 U/mL), pH 8 (0.274 U/mL), pH 3 (0.136 U/mL), and pH 4 (0.104 U/mL). The original pH of the media before pH adjustment and autoclaving was 6.3. The higher pectinase activity produced was found in the media at pH 5–7. The effect of cultivation temperature on pectinase production was performed at pH 6, a shaking speed of 100 rpm, 0.5% WBP (C source), and a medium/flask volume ratio of 100/250 mL. The pectinase activities produced at different temperatures were all highest on the fourth day in the order of 37 °C (0.754 U/mL), 40 °C (0.734 U/mL), 45 °C (0.668 U/mL), 30 °C (0.486 U/mL), and 50 °C (0.035 U/mL). The effect of shaking speed on pectinase production was performed at pH 6 and 37 °C using 0.5% WBP (C source). The pectinase activities produced in different shaking speeds were all highest on the fourth day in the order of 100 rpm (0.715 U/mL), 150 rpm (0.706 U/mL), 200 rpm (0.680 U/mL), and 50 rpm (0.303 U/mL). The culture conditions for pectinase production by *B. amyloliquefaciens* TKU050 before and after the optimized study are summarized in Table 1. The pectinase productivity was increased from 0.11 U/mL to 0.76 U/mL after the optimized investigation. From the above study, it could be deduced that *B. amyloliquefaciens* TKU050 was able to produce an appreciable amount of pectinase using wheat bran and could be considered as a potential candidate for industrial applications such as valorization of wastes [25], clarification of wine and fruit juices [13,14], preparation of prebiotic [26], degradation of biomass to produce biofuel [27], etc.

### 3.4. Purification of the Pectinase

**Figure 2:**
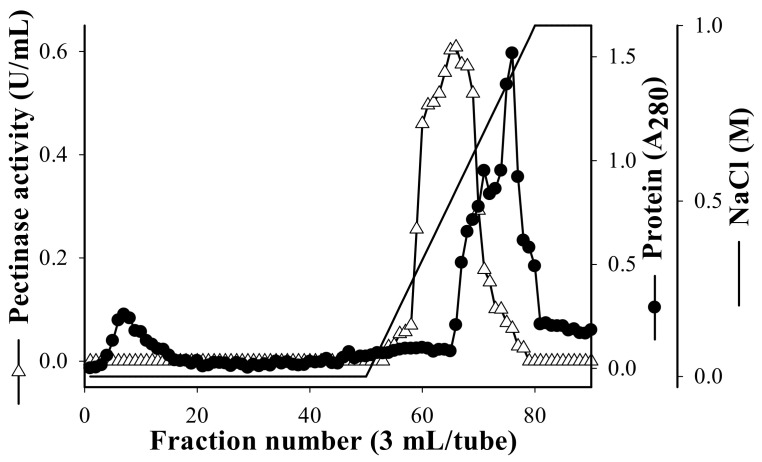
Elution profile of *B. amyloliquefaciens* TKU050 pectinase in a High Q column.

To date, there have been few reports of the pectinase being produced by *B. amyloliquefaciens* strains. The pectinase production of *B. amyloliquefaciens* SW106 has been studied; however, its purification process was not performed [28]. To compare the characteristics of the pectinase produced by *B. amyloliquefaciens* TKU050 against other reported strains, the pectinase of this strain was purified from the four-day culture supernatant by a series of steps. The protein was precipitated from the culture supernatant by ammonium sulfate (80% *w*/*v*) and eluted by ion-exchange chromatography using a Macro-Prep High Q column with a linear gradient of 0–1 M NaCl (Figure 2). The eluted peaks of the pectinase fractions were pooled and desalted for further purification by high-performance liquid chromatography (HPLC) with a KW-802.5 column.

**Figure 3:**
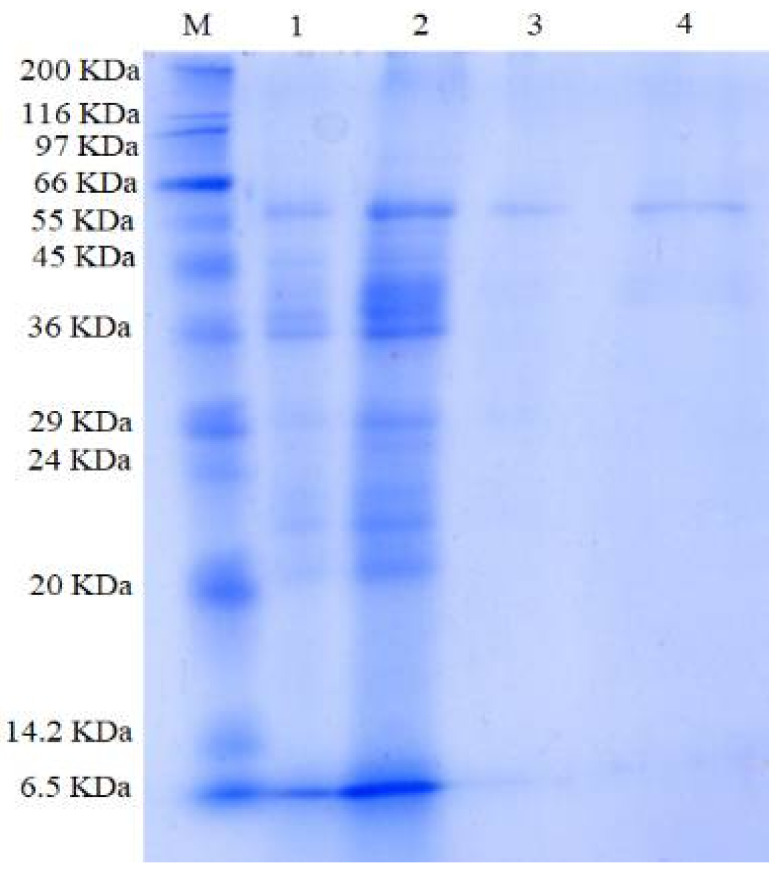
SDS-PAGE profiles of *B. amyloliquefaciens* TKU050 pectinase: (1) culture supernatant; (2) crude enzyme after (NH_4_)_2_SO_4_ precipitation; (3) pectinase fraction after running FPLC with a Macro-prep High Q column; (4) purified pectinase after running HPLC with a KW802.5 column. M represents the protein markers.

After isolation and purification, approximately 54.9 mg of *B. amyloliquefaciens* TKU050 pectinase was obtained. The recovery yield of the obtained TKU050 pectinase was 7.2% with 19.8-folds of specific activity (Table 2). The molecular weight of the TKU050 pectinase was approximately 58 kDa, as determined by the SDS-PAGE method (Figure 3). *Bacillus* pectinases have molecular weights ranging from 37 to 66 kDa except for *Bacillus* sp. DT7 (Table 3).

### 3.5. Properties of TKU050 Pectinase

#### 3.5.1. Effects of Temperature and pH

**Figure 4:**
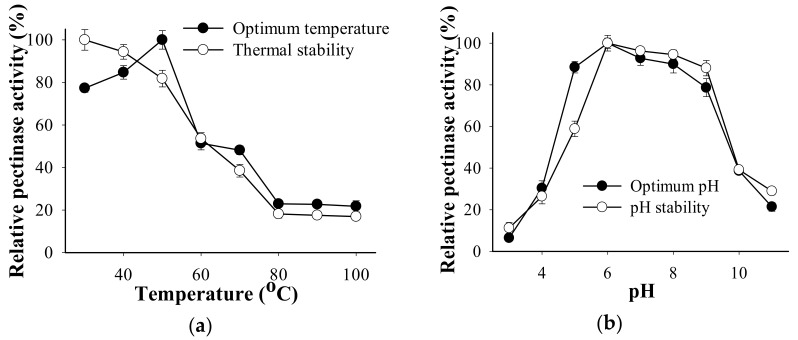
Effects of temperature and pH on the activity and stability of *B. amyloliquefaciens* TKU050 pectinase: (**a**) temperature; (**b**) pH. All data points are the mean and standard deviation.

The thermal stability of *B. amyloliquefaciens* TKU050 pectinase was tested by treating the enzyme solution at different temperatures for one h. The optimal temperature for *B. amyloliquefaciens* TKU050 pectinase was found to be 50 °C (Figure 4a). *B. amyloliquefaciens* TKU050 pectinase retained above 80% of the initial activity until 50 °C and then gradually lost its activity at higher temperatures. Pectinase from *Bacillus* strains has also been found to work best at temperatures ranging from 40 °C to 60 °C [16,29,32,33].

The pH activity profile of *B. amyloliquefaciens* TKU050 pectinase is shown in Figure 4b. The optimum enzyme activity was observed at pH 6 and was stable at the pH range of pH 6 to pH 8. In earlier studies, the optimum pH of pectinase from *Bacillus* strains was commonly reported at neutral or alkaline values, such as *B. subtilis* CM5 (pH 7) [32], *Bacillus* sp. AD1 (pH 7) [33], *B. subtilis* Btk27 (pH 7.5) [16], and *Bacillus* sp. DT7 (pH 8) [29].

#### 3.5.2. Effects of Divalent Metal Ions, EDTA, and Surfactants

Table 4 illustrates the activity of *B. amyloliquefaciens* TKU050 pectinase incubated with various chemicals. EDTA and majority of the tested divalent metal ions (Zn^2+^, Fe^2+^, Mn^2+^, Mg^2+^, and Ba^2+^) had no impact on pectinase activity except for Ca^2+^, and Cu^2+^. Surfactants (Triton X-100, Tween 20, Tween 40, and SDS) were also tested for their effect on *B. amyloliquefaciens* pectinase activity. A slight enhancing effect was found using 0.5% of the nonionic surfactants Triton X-100 (117.48%) and 0.5% Tween 40 (131.94%), whereas the ionic surfactant SDS remarkably inhibited the pectinase activity (retained 20.56%). The activating effect of Triton X-100 and Tween 40 may be related to the capacity of surface-active reagents to enhance the frequency of contact between the pectin and the pectinase active site [43]. The suppressing effect of SDS on the pectinase activity could be attributed to its capacity to alter the pectinase secondary structure [44].

#### 3.5.3. Substrate Specificity

**Figure 5:**
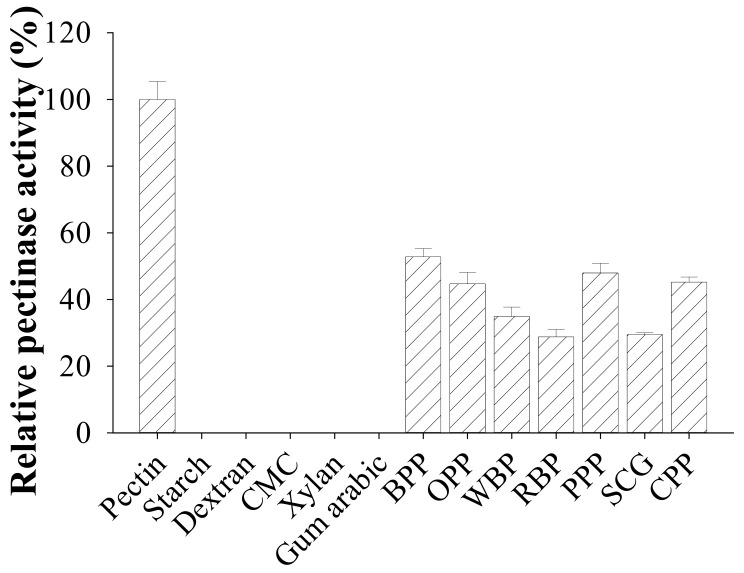
Substrate specificity of *B. amyloliquefaciens* TKU050 pectinase, including CMC (Carboxymethyl Cellulose), BPP (banana peel powder), OPP (orange peel powder), WBP (wheat bran powder), RBP (rice bran powder), PPP (pomelo peel powder), SCG (spent coffee grounds), and CPP (coffee pulp powder). All error bars are the ±SD (standard deviation) of three different experiments.

As shown in Figure 5, *B. amyloliquefaciens* TKU050 pectinase expressed the greatest activity in the order of pectin > banana peel powder (BPP) > pomelo peel powder (PPP) > coffee pulp powder > wheat bran powder (WBP) > spent coffee grounds (SCG) > rice bran powder (RBP). No activity was found against starch, dextran, carboxymethyl cellulose (CMC), xylan, or gum arabic. This result suggested that *B. amyloliquefaciens* TKU050 pectinase could exhibit pectolytic ability compared to other enzyme activities, such as amylases, xylanases, and cellulases.

**Figure 6:**
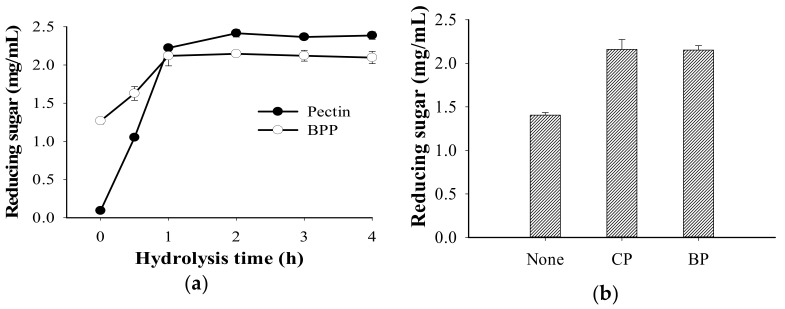
The hydrolysis of BPP and pectin catalyzed by TKU050 pectinase (**a**); BPP catalyzed by commercial pectinase and TKU050 pectinase (**b**). BPP, banana peel powder; CP, commercial pectinase from *A. niger*; and BP, *B. amyloliquefaciens* TKU050 pectinase. All error bars are the ±SD (standard deviation) of three different experiments.

Based on the results shown in Figure 5, banana peel powder (BPP) was observed to be the most suitable substrate next to pectin for the TKU050 pectinases; therefore, their hydrolysis was conducted and analyzed via the amount of reducing sugars released during the reaction period. The pectinases from the culture supernatant (60 mL) were used to catalyze the hydrolysis of BPP (1% *w*/*v*) and pectin (1% *w*/*v*) in a 50 mM sodium phosphate buffer (pH 7). As shown in Figure 6a, the rate of BPP hydrolysis was high within the first hour (from 1.25 mg/mL at 0 h to 2.2 mg/mL at 1 h) and remained stationary thereafter. A similar hydrolysis pattern was observed in pectin, as the hydrolysis rate increased dramatically with the first hour (from 0.1 mg/mL at 0 h to 2.3 mg/mL at 1 h), reached 2.4 mg/mL at the second hour, and remained the same thereafter. In a further experiment, the BPP-hydrolyzing ability of *B. amyloliquefaciens* TKU050 pectinase was also compared to a commercial pectinase from *Aspergillus niger*. The result showed that the amount of reducing sugar released from the BPP-hydrolysis showed no significant difference between *B. amyloliquefaciens* pectinase (2.15 mg/mL) and *A. niger* pectinase (2.16 mg/mL), indicating that *B. amyloliquefaciens* TKU050 pectinase could be a potential enzyme for hydrolyzing BPP (Figure 6b).

### 3.6. Evaluation of the Growth Enhancing Effect of BPP Hydrolysates on Lactic Acid Bacteria

**Figure 7:**
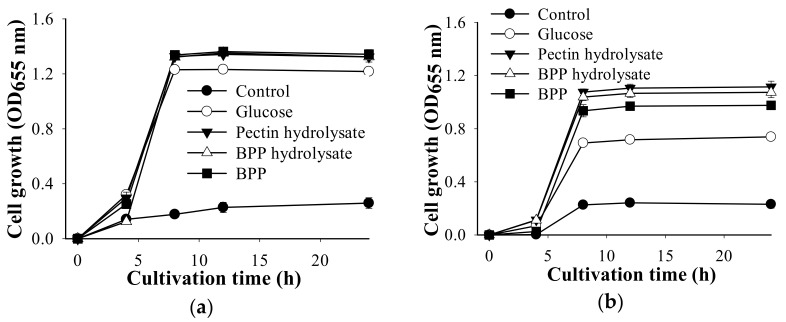

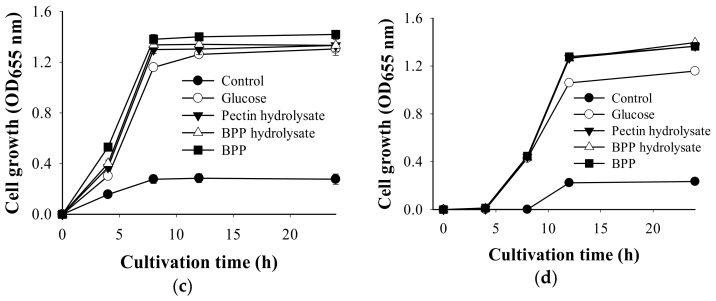
Effect of BPB hydrolysate, pectin hydrolysate, BPB, and glucose on the growth of lactic acid bacteria strains: (**a**) *L. paracasei* subsp. *paracasei* BCRC14023; (**b**) *L. rhamnosus* BCRC16000; (**c**) *L. rhamnosus* BCRC10494; (**d**) *L. lactis* BCRC10791. The error bars represent standard deviations (*n* = 3).

The effect of the hydrolysates obtained from the hydrolysis of BPP and the pectin generated by TKU050 pectinases were studied for their prebiotic effects on four lactic acid bacteria: *L. paracasei* subsp. *paracasei* BCRC14023 (Figure 7a), *L. rhamnosus* BCRC16000 (Figure 7b), *L. rhamnosus* BCRC10494 (Figure 7c), and *L. lactis* BCRC10791 (Figure 7d). For comparison, BPP and glucose were also investigated. As shown in Figure 7, the four tested supplemented samples all showed enhancing effect on the tested four lactic acid bacteria. Among them, the BPP hydrolysates, pectin hydrolysates, and BPP had better prebiotic effects than that of glucose. The BPP hydrolysates gained a higher enhancing effect than that of BPB on the growth of *L. rhamnosus* BCRC16000 (Figure 7b). These results suggested that BPP and its hydrolysates produced by TKU050 pectinases may have the potential to become prebiotic candidates.

## 4. Conclusions

Pectinase is considered to be an effective tool for preparing pectin oligosaccharides, but the enzyme’s cost is a possible stumbling block for this application. Thus, the objective of the current study was to post a cost-effective and environmentally friendly bioprocessing of pectin-containing by-products for pectinase production by *B. amyloliquefaciens* TKU050. Following that, a 58 kDa pectinase was purified from the wheat bran culture medium. Additionally, the hydrolysis product of banana peel powder catalyzed by this enzyme showed prebiotic effects on the four tested lactic acid bacteria, indicating that *B. amyloliquefaciens* TKU050 pectinase could be a good candidate for producing prebiotics.

## Figures and Tables

**Table 1:** The optimized culture conditions for the production of pectinase by *B. amyloliquefaciens* TKU050.

Compared Factors	Before Optimization	After Optimization
Culture time (day)	3	4
C concentration (*w*/*v*)	1% pectin	0.5% WBP
Cultivation temperature (°C)	37	37
pH value	6.6	6.0
Shaking speed (rpm)	150	100
Pectinase activity (U/mL)	0.11	0.76

**Table 2:** Purification of *B. amyloliquefaciens* TKU050 pectinase.

Step	Total Protein (mg)	Total Activity (U)	Specific Activity (U/mg)	Recovery (%)	Purification (fold)
Cultural supernatant	15,003.63	689.06	0.046	100.00	1.00
(NH_4_)_2_SO_4_ precipitation	2136.57	566.83	0.265	82.26	5.76
Ion-exchange chromatography	396.50	319.87	0.807	46.42	17.54
Gel filtration	54.90	49.89	0.909	7.24	19.79

**Table 3:** Comparison of pectinase produced by *Bacillus* strains.

Strain	MW (KDa)	Opt. Temp. (°C)	Opt. pH	Carbon Source	Reference
*B. amyloliquefaciens* TKU050	58	50	6	wheat bran	This study
*Bacillus* sp. DT7	106	60	8	pectin	[29]
*B. paralicheniformis* CBS3	53	60	9.1	pectin	[30]
*Bacillus sp. MBRL576*	66	-	-	sucrose	[31]
*B. subtilis* Btk27	-	50	7.5	wheat bran	[16]
*B. subtilis*	38	-	-	pectin	[4]
*B. subtilis* CM5	56	50	7	cassava bagasse	[32]
*Bacillus* sp. AD 1	-	37	7	pectin	[33]
*B. tequilensis* CAS-MEI-2-33	45.4	40	10	tobacco stalk	[12]
*B. sonorensis* MPTD1	-	-	-	pectin	[34]
*Bacillus* sp. MFW7	37	-	-	cassava waste	[35]
*B. subtilis* TYg4-3	-	-	-	lactose	[28]
*B. amyloliquefaciens* SW106	-	-	-	maltose	[28]
*B. subtilis* SS	-	-	-	wheat bran	[36]
*Bacillus* sp. Y1	-	-	-	starch, wheat bran, and sucrose	[37]
*B. subtilis* ZGL14	65	50	8.6	starch	[38]
*B. licheniformis* KIBGE-IB21	-	-	-	wheat bran	[39]
*B. pumilus* dcsr1				sesame oilseed cake	[40]
*B. mojavensis* I4	-	60	8	carrot peel	[41]
*B. licheniformis* KIBE-IB3	-	-	-	wheat bran	[42]
*B. safensis* M35	-	-	-	wheat bran and citrus peel	[7]
*B. altitudinis* J208	-	-	-	wheat bran and citrus peel	[7]

**Table 4:** Effects of various chemicals on the activity of *B. amyloliquefaciens* TKU050 pectinase. All error bars are the ±SD (standard deviation) of three different experiments.

Metal Ion/Surfactant	Concentration	Relative Pectinase Activity (%)
None		100 ± 2.29
Zn^2+^	2 mM	80.83 ± 3.38
Fe^2+^	2 mM	96.75 ± 6.03
Mn^2+^	2 mM	97.47 ± 1.21
Cu^2+^	2 mM	26.35 ± 0.24
Mg^2+^	2 mM	102.29 ± 1.93
Ba^2+^	2 mM	87.46 ± 4.94
Ca^2+^	2 mM	114.71 ± 2.53
EDTA	2 mM	106.99 ± 3.74
Triton X-100	0.5%	117.48 ± 2.41
Tween 20	0.5%	96.75 ± 2.41
Tween 40	0.5%	131.94 ± 8.2
SDS	2 mM	20.56 ± 0.72

## Data Availability

The data presented in this study are available on request from the corresponding author.
